# Involvement of hepatic macrophages in the antifibrotic effect of IGF-I-overexpressing mesenchymal stromal cells

**DOI:** 10.1186/s13287-016-0424-y

**Published:** 2016-11-22

**Authors:** Esteban Fiore, Mariana Malvicini, Juan Bayo, Estanislao Peixoto, Catalina Atorrasagasti, Romina Sierra, Marcelo Rodríguez, Sofia Gómez Bustillo, Mariana G. García, Jorge B. Aquino, Guillermo Mazzolini

**Affiliations:** 1 Gene Therapy Laboratory, Facultad de Ciencias Biomédicas, Universidad Austral, Av. Pte. Peron 1500, Derqui-Pilar, Buenos Aires B1629AHJ Argentina; 2 CONICET (Consejo Nacional de Investigaciones Científicas y Técnicas) Godoy Cruz 2290, Buenos Aires, Argentina; 3 Developmental Biology and Regenerative Medicine Laboratory, Facultad de Ciencias Biomédicas, Universidad Austral, Av. Pte. Peron 1500, Derqui-Pilar, Buenos Aires B1629AHJ Argentina

**Keywords:** MSCs, Liver macrophages, Cirrhosis, Insulin-like growth factor I, Hepatic stellate cells, DNA repair

## Abstract

**Background:**

Cirrhosis is a major health problem worldwide and new therapies are needed. Hepatic macrophages (hMø) have a pivotal role in liver fibrosis, being able to act in both its promotion and its resolution. It is well-known that mesenchymal stromal cells (MSCs) can modulate the immune/inflammatory cells. However, the effects of MSCs over hMø in the context of liver fibrosis remain unclear. We previously described evidence of the antifibrotic effects of in vivo applying MSCs, which were enhanced by forced overexpression of insulin-like growth factor 1 (AdIGF-I-MSCs). The aim of this work was to analyze the effect of MSCs on hMø behavior in the context of liver fibrosis resolution.

**Methods:**

Fibrosis was induced in BALB/c mice by chronic administration of thioacetamide (8 weeks). In vivo gene expression analyses, in vitro experiments using hMø isolated from the nonparenchymal liver cells fraction, and in vivo experiments with depletion of Mø were performed.

**Results:**

One day after treatment, hMø from fibrotic livers of MSCs-treated animals showed reduced pro-inflammatory and pro-fibrogenic gene expression profiles. These shifts were more pronounced in AdIGF-I-MSCs condition. This group showed a significant upregulation in the expression of arginase-1 and a higher downregulation of iNOS expression thus suggesting decreased levels of oxidative stress. An upregulation in IGF-I and HGF expression was observed in hMø from AdIGF-I-MSCs-treated mice suggesting a restorative phenotype in these cells. Factors secreted by hMø, preconditioned with MSCs supernatant, caused a reduction in the expression levels of hepatic stellate cells pro-fibrogenic and activation markers. Interestingly, hMø depletion abrogated the therapeutic effect achieved with AdIGF-I-MSCs therapy. Expression profile analyses for cell cycle markers were performed on fibrotic livers after treatment with AdIGF-I-MSCs and showed a significant regulation in genes related to DNA synthesis and repair quality control, cell cycle progression, and DNA damage/cellular stress compatible with early induction of pro-regenerative and hepatoprotective mechanisms. Moreover, depletion of hMø abrogated such effects on the expression of the most highly regulated genes.

**Conclusions:**

Our results indicate that AdIGF-I-MSCs are able to induce a pro-fibrotic to resolutive phenotype shift on hepatic macrophages, which is a key early event driving liver fibrosis amelioration.

**Electronic supplementary material:**

The online version of this article (doi:10.1186/s13287-016-0424-y) contains supplementary material, which is available to authorized users.

## Background

Cirrhosis is characterized by the excessive accumulation of collagen and other extracellular matrix proteins, leading to liver function impairment [1]. It has no other effective treatments than liver transplantation [1, 2] but, due to the scarcity of donors, new therapeutic approaches are urgently needed.

Hepatic stellate cells (HeSCs) play a key role in liver fibrogenesis [1]. After injury these cells get activated; they then proliferate, migrate to the injured areas, and produce large amounts of extracellular matrix proteins and pro-fibrogenic cytokines such as transforming growth factor beta 1 (TGF-β1) or platelet-derived growth factor (PDGF) [3]. Hepatic macrophages (hMø) composed by Kupffer cells and infiltrating macrophages, are also involved in liver fibrogenesis [4]. They secrete factors such as TGF-β1 and PDGF, which are known to induce HeSCs activation [5]. They also release tumor necrosis factor alpha (TNF-α), interleukin-1β (IL-1β) and other chemokines causing further hepatocellular damage due to inflammatory exacerbation and oxidative stress [6, 7]. However, recent literature also involved hMø in liver fibrosis resolution, through increased production of matrix metalloproteinases (MMPs) and/or the induction of activated HeSCs apoptosis [7].

Mesenchymal stromal cells (also known as mesenchymal stem cells; MSCs) are immune privileged multipotent progenitors [8], able to modulate immune/inflammatory responses [9] and to migrate to injury sites [10]. The application of either autologous or allogeneic MSCs resulted in the amelioration of disease conditions. Many clinical trials have studied the therapeutic effect of MSCs in patients with cirrhosis with promising results. In general, clinical studies showed an improvement in the quality of life and liver function or prognostic indicators such as improvement in the model for end-stage liver disease and Child-Pugh scores [11]. In addition, systemic administration of MSCs was found to ameliorate liver fibrosis in mice; moreover, their use as vehicles of therapeutic genes was proposed as a potential strategy to enhance such outcome improvements [12]. Nevertheless, there is little knowledge regarding in vivo mechanisms therein involved, which were, in most cases, analyzed at several days or even weeks after treatment and would likely be therefore indirect. Early events after MSCs applications would help explain experimental outcomes and could be crucial to improve fibrosis resolution. We have previously shown that MSCs engineered to exogenously produce adenovirus containing insulin-like growth factor I MSCs (AdIGF-I-MSCs) were able to further reduce liver fibrosis degree when compared to MSCs alone, in thioacetamide (TAA) chronic intoxication and bile duct ligation models [13]. The day after AdIGF-I-MSCs infusion, a significant increase in the number of proliferating hepatocytes was observed as well as an upregulation in endogenous IGF-I, hepatocyte growth factor (HGF), proliferating cell nuclear antigen (PCNA) and TNF-related weak inducer of apoptosis (TWEAK; a marker related to progenitor cells) expression levels. A reduction in the activation of HeSCs was only found at the third day after treatment [13]. However, it is not yet clear whether MSCs would induce such early mechanisms acting directly over hepatocytes and HeSCs or if some other cellular populations might also be involved. Interestingly, it has been previously reported in other degenerative diseases that MSCs are able to induce a macrophages phenotype switch [14, 15], but at the moment this has not been analyzed in the context of liver fibrosis.

In this study, we asked whether or not changes in the hMø population might mediate the beneficial effects of MSCs in an experimental in vivo model of advanced liver fibrosis. We herein show a shift in hMø, from a pro-inflammatory to an anti-inflammatory/restorative profile, with an upregulation of IGF-I and HGF expression levels, at 1 day after treatment with AdIGF-I-MSCs. In addition, we also show that factors released by hMø which were preconditioned with MSCs supernatants were able to reduce the HeSCs activation status. Furthermore, the therapeutic outcome of AdIGF-I-MSCs infusion was significantly abrogated when the liver was depleted from macrophages. Finally, hMø were found to be involved in the pro-regenerative mechanisms induced by MSCs. Our results strongly suggest that hMø are involved in the early events leading to the reduction of HeSC activation and fibrosis amelioration after MSCs treatment.

## Methods

### Cell culture and adenoviral transfection of mouse bone marrow MSCs

MSCs isolation and characterization procedures as well as adenoviral vectors and transfection procedures were previously described (see Additional file 1) [13]. Conditioned media from MSCs were prepared as follows: the same number of cells were seeded at 70% of confluence and infected with adenovirus containing green fluorescent protein (AdGFP) or AdIGF-I. Three days after infection, AdIGF-I-MSCs and AdGFP-MSCs were extensively washed and serum starved. After 24 hours, supernatant from infected MSCs were collected, centrifuged at 5000 × g for 10 min at 4 °C and stored for further analysis.

### In vivo experimental design

Six-to-eight-week-old male BALB/c mice were purchased from CNEA (Comisión Nacional de Energía Atómica, Ezeiza, Buenos Aires, Argentina). Animals were maintained at our Animal Resources Facility (Faculty of Biomedical Sciences, Austral University) in accordance with the experimental ethics committee and the National Institutes of Health (NIH) guidelines on the ethical use of animals. Fibrosis was induced by intraperitoneal (i.p.) administration of 0.2 mg/g bodyweight of thioacetamide (TAA) (Sigma-Aldrich, St Louis, MO, USA), three times per week, for 6 weeks. Animals were then sacrificed for hepatic macrophage isolation or intravenously (i.v.) injected (tail) with saline, AdGFP-MSCs or AdIGF-I-MSCs (5 × 10^5^ cells/animal; n = 5/condition). The day after saline/MSCs treatments, animals were sacrificed and liver samples were dissected out and used for subsequent studies or for hMø isolation (Additional file 1: Figure S1a).

### In vivo hepatic macrophages quantification

Hepatic macrophages quantification in frozen liver sections was performed by immunofluorescence for F4/80. After a 1-h incubation in blockage buffer (5% normal donkey serum, Jackson ImmunoResearch, West Grove, PA, USA; 1% BSA, 0.3% Triton-X in PBS; room temperature), tissue was incubated overnight at 4 °C with a rabbit anti-F4/80 (F4/80; 1/400; Abcam, Cambridge, MA, USA). After extensive washing, tissue was incubated with FITC-conjugated donkey anti-rabbit IgG secondary antibodies (room temperature; Vector Laboratories, Inc., Burlingame, CA, USA). Slides were mounted in mounting media with DAPI (Vector Laboratories, Inc.) and analyzed under a fluorescence microscope. Control experiments without primary antibody showed only a faint background staining (not shown). Images were taken with direct fluorescent microscopy (200×; 50 images/condition) with green (FITC-F4/80-positive cells) and red (autofluorescent cells) filters. Merged images were analyzed using the ImageJ software (National Institutes of Health, Bethesda, MD, USA) and autofluorescent red cells were excluded. F4/80-positive cells were counted using CellProfiler software (www.cellprofiler.com). Scores are expressed as mean number of cells/field.

### Hepatic macrophages isolation

For in vitro experiments, the hMø-enriched fraction was obtained as previously described [16]. Briefly, BALB/c mice were sacrificed and liver was digested with collagenase (Sigma-Aldrich) through local perfusion. The nonparenchymal cell fraction was separated by a density gradient with Histodenz (Sigma-Aldrich) 30% in PBS from total liver cells. Equal numbers of cells were seeded into the wells. After 20 min of incubation in a 12-well plate of the cell suspension obtained, hMø remained adhered to plastic. Then, they were collected with Trizol reagent (Sigma-Aldrich) from RNA extraction or maintained in culture for subsequent studies. To confirm hMø purity, cells were immunostained with a rat anti-mouse F4/80 antibody (Abcam, Cambridge, MA, USA) 1:350 in PBS for 45 min at 4 °C. After three washes with 1%BSA/PBS, cells were incubated with a fluorescein isothiocyanate anti-rat (Vector Laboratories, Inc.) antibody 1:100 in PBS for 45 min at 4 °C. Then, cells were observed under a fluorescence microscope (Nikon Eclipse E800, Nikon, Tokyo, Japan).

### In vitro hMø experiments

Macrophages obtained from fibrotic livers 1 day after saline/cellular treatments were collected in Trizol reagent (Sigma-Aldrich) for RNA extraction, or medium was removed and cells maintained in serum-starved Dulbecco’s modified Eagle’s medium (DMEM) for 24 h. In the latter case, cells supernatants were harvested and centrifuged 10 min at 5000 × g at 4 °C for protein level analysis by enzyme-linked immunosorbent assay (ELISA) (Additional file 1: Figure S1a).

Liver macrophages from fibrotic mice without saline/cellular treatments were obtained after 6 weeks of TAA injection. They were incubated for 18 h with DMEM (control) or conditioned media from AdIGF-I-MSCs or AdGFP-MSCs supplemented with 2% fetal bovine serum (FBS) (Additional file 1: Figure S1b). Then, cells were washed and collected with Trizol reagent (Sigma-Aldrich) or maintained in culture with DMEM without serum for an additional 24 hours, harvested and centrifuged at 5000 × g for 10 min at 4 °C for protein levels analysis by ELISA or subsequent experiments.

### Hepatic stellate cell cultures

The CFSC-2G hepatic stellate cell line, originally established from cirrhotic rat liver, was kindly provided by Dr. Marcos Rojkind (Albert Einstein College of Medicine, New York, NY, USA). Cells were cultured in MEM (Invitrogen, Life Technologies, Waltham, MA, USA) supplemented with 10% FBS (Invitrogen) and nonessential amino acids. To evaluate the effect of factors released by hMø preconditioned with MSC supernatants on the activity of HeSCs, CFSC-2G cells were incubated for 18 hours with supernatants of hMø preincubated with DMEM (control), AdGFP-MSC or AdIGF-I-MSC conditioned media (as described before). Then, cells were detached, washed and immersed in Trizol reagent (Sigma-Aldrich) for total RNA extraction. Levels of TGF-β1, alpha-smooth muscle actin (α-SMA) and collagen 1A2 (COL1A2) mRNA expression were determined by real-time polymerase chain reaction (qPCR) (Additional file 1: Figure S1b).

### Depletion of hepatic macrophages

Liver fibrosis was induced in BALB/c mice by intraperitoneal administration of TAA, three times per week, for 8 weeks. On week 6, hepatic macrophages were depleted by intravenous injection of liposome-encapsulated clodronate (LipClod) (0.2 ml/25 g bodyweight) [17] or saline solution (nondepleted control mice) (n = 12 per group). One day later, saline or AdIGF-I-MSCs were intravenously injected to each group (n = 6/condition). At 8 weeks, animals were sacrificed and liver samples were dissected out and used for Sirius Red analyses or RNA extraction (Additional file 1: Figure S1c).

### Cell cycle qPCR array

Total RNA sample was isolated using an RNeasy Minikit protocol (Qiagen, Hilden, Germany) from the liver of saline or AdIGF-I-MSC-treated mice 1 day after systemic administration. Equal amounts of RNA from different samples were then pooled and reverse-transcribed to cDNA. The quality of the cDNA conversion was confirmed by PCR on pooled samples, using glyceraldehyde 3-phosphate dehydrogenase (GAPDH) primers. Expression levels of 84 genes involved in the cell cycle were evaluated using the RT2 profiler PCR array system (catalog number: PAMM-020Z; SABiosciences, Frederick, MD, USA) following the manufacturer’s instructions. The mRNA expression levels obtained for each gene were normalized to the expression of the GAPDH housekeeping gene. Finally, data results were analyzed by the manufacturer’s online website and scores were expressed as fold change versus saline group (fold change: ΔΔCt).

### Statistical analyses

Data are expressed as mean ± SEM. Statistical analyses were performed using Student’s *t* test or ANOVA according to data distribution. Differences were considered to be significant when *p* < 0.05. All experiments were performed at least three times in triplicate (Additional file 1).

## Results

### Enhanced accumulation of macrophages in the fibrotic liver after MSCs transplantation

**Fig. 1:**
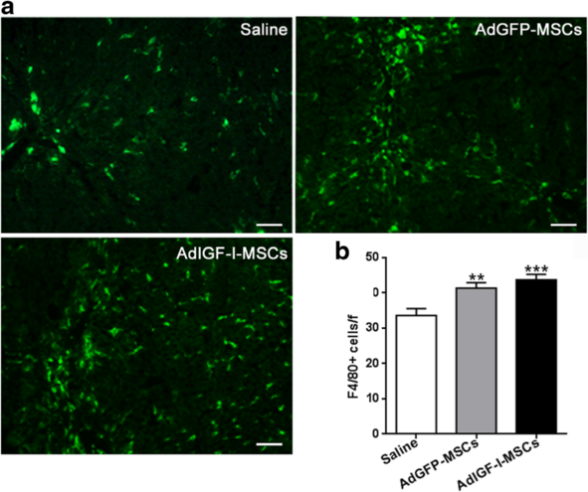
Incidence of macrophages in fibrotic liver tissue after MSC treatments. **a** Representative microphotographs of liver sections stained for F4/80, at 1 day after saline, AdGFP-MSCs or AdIGF-I-MSCs administration. **b** Quantitative graph of F4/80-positive cells in liver sections. Bars represent the average of positive cells/field ± SEM from ten representative visual fields. ANOVA Tukey’s post-test, ^**^ *p* < 0.01 and ^***^ *p* < 0.001 vs. saline-treated mice

We previously showed that MSCs can be efficiently transduced with recombinant adenovirus with sustained significant levels of transgene expression up to 8 days post-infection. No significant changes in MSCs immunological properties were found after their genetic manipulation. Moreover, systemic administration of AdIGF-I-MSCs further reduced the collagen deposition and the degree of HeSCs activation. Considering the well-known immune-modulatory properties of MSCs, we asked whether they might induce changes in numbers of hMø in a TAA chronic model of hepatic fibrosis. Indeed, increased numbers of F4/80^+^ macrophages were found in liver sections from mice treated with AdGFP-MSCs and AdIGF-I-MSCs when compared to vehicle, at 1 day after treatment (Fig. 1a,b).

### AdIGF-I-MSCs modulate the gene expression profile of hepatic macrophages in advanced liver fibrosis

**Fig. 2:**
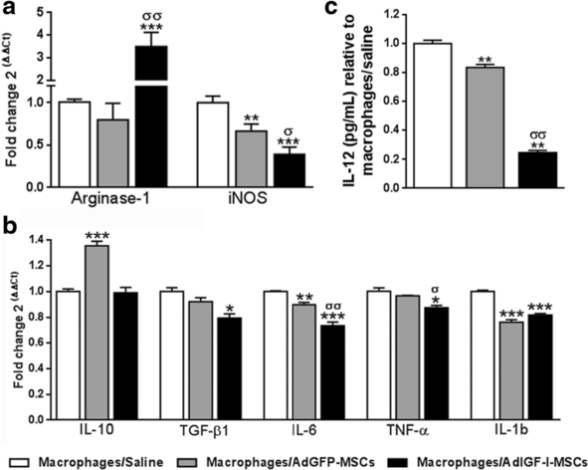
In vivo modulation of hepatic macrophages expression profile by MSCs. mRNA expression levels of the nitric oxide production mediators arginase-1 and iNOS (**a**) and IL-10 and pro-fibrogenic cytokines TGF-β1, IL-6, TNF-α, and IL-1bβ (**b**) in macrophages at 1 day after saline (*white bars*), AdGFP-MSCs (*gray*) and AdIGF-I-MSCs (*black*) treatments. **c** IL-12 protein levels in hepatic macrophages supernatants relative to macrophages/saline condition. ANOVA Tukey’s post-test, ^*^ *p* < 0.05; ^**^ *p* < 0.01 and ^***^ *p* < 0.001 vs. macrophages/saline condition; ^σ^ *p* < 0.05 and ^σσ^ *p* < 0.01 vs. macrophages/AdGFP-MSCs condition

Considering that factors secreted by AdIGF-I-MSCs were shown to induce IGF-I and HGF expression in hepatocytes and that IGF-I is known to counteract TGF-β1 signaling [18], we next asked whether MSCs might induce changes in relevant genes involved in macrophages activity in vivo. For this purpose, enriched fractions of hMø, with more than 90% of cells being F4/80^+^ [16], were obtained at 1 day after saline/cellular treatments (see Additional file 1: Figure S1a). Interestingly, systemic administration of AdIGF-I-MSCs induced significant changes in the mRNA expression levels of enzymes involved in nitric oxide (NO) production in acutely isolated hMø from fibrotic livers (Fig. 2a). For instance, arginase-1 was upregulated after AdIGF-I-MSCs when compared to controls (Fig. 2a). In addition, iNOS was downregulated in hMø isolated from AdGFP-MSCs-treated mice when compared to vehicle, and its mRNA levels were further reduced in AdIGF-I-MSCs condition. Moreover, the mRNA expression of pro-inflammatory cytokines, such as TNF-α, IL-6 and IL-1β, was significantly downregulated after AdIGF-I-MSCs treatment when compared to vehicle (Fig. 2b). Furthermore, TNF-α and IL-6 mRNA expression levels were downregulated in the AdIGF-I-MSCs group when compared to control MSCs (Fig. 2b). However, hMø IL-10 mRNA expression levels were upregulated only after AdGFP-MSCs treatment. In addition, reduced IL-12 protein levels were found in the supernatant of AdGFP-MSCs hMø when compared to vehicle, and such changes were much more remarkable in AdIGF-I-MSCs hMø condition (Fig. 2c). It is worth noting that mRNA expression levels of TGF-β1, an anti-inflammatory but pro-fibrogenic cytokine, were significantly reduced in AdIGF-I-MSCs hMø in comparison with vehicle and AdGFP-MSCs groups. Altogether, these results suggest that in vivo applications of AdGFP-MSCs and AdIGF-I-MSCs differentially regulate the expression profile of hMø in the TAA liver fibrosis model.

### Soluble factors secreted by AdIGF-I-MSCs modulate the expression profile of hepatic macrophages in the context of liver fibrosis

**Fig. 3:**
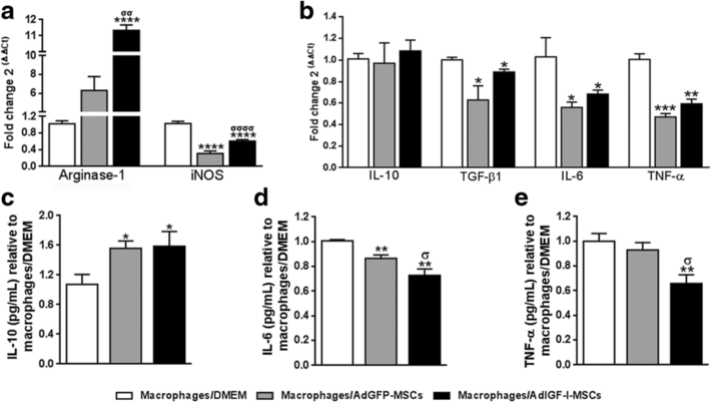
MSCs secrete factors which modulate the hepatic macrophages phenotype. mRNA expression levels of nitric oxide production mediators arginase-1 and iNOS (**a**) and of cytokines involved in the fibrogenic process (**b**) in macrophages preincubated 18 hours with DMEM (*white bars*), or AdGFP-MSCs (*gray*) or AdIGF-I-MSCs (*black*) supernatants. Similar comparisons were done at the protein level for IL-6 and IL-10 (supernatants; **c** and **d**, respectively) and TNF-α (cytoplasmic extract; **e**). ANOVA Tukey’s post-test; ^*^ *p* < 0.05, ^**^ *p* < 0.01, ^***^ *p* < 0.001 and ^****^ *p* < 0.0001 vs. macrophages/DMEM; ^σ^ *p* < 0.05, ^σσ^ *p* < 0.01 and ^σσσσ^ *p* < 0.0001 vs. macrophages/AdGFP-MSCs

In order to evaluate if the previously shown effects of MSCs could be explained by paracrine mechanisms (mediated by soluble factors acting at short range), hMø were isolated from fibrotic livers (after 6 weeks of TAA treatment) and were incubated for 18 hours with conditioned media from AdIGF-I-MSCs or AdGFP-MSCs cultures or with DMEM (see Additional file 1: Figure S1b). Cells were washed and collected, or medium was replaced by serum-starved DMEM for 24 h and supernatant/cells collected. Consistent with previous results, mRNA expression levels of arginase-1 were increased in hMø incubated with AdIGF-I-MSCs supernatant when compared to controls, while treatments with both MSCs supernatants resulted in downregulation of iNOS gene expression (Fig. 3a). Furthermore, TGF-β1, IL-6, and TNF-α mRNA levels were also reduced in hMø after incubation with MSCs supernatants when compared to DMEM, whereas IL-10 gene expression remained unaffected (Fig. 3b). However, IL-10 secreted protein levels were increase in supernatants of hMø pretreated with MSCs conditioned media, when compared to DMEM control (Fig. 3c). Interestingly, levels of IL-6 and TNF-α proteins were reduced in Ad-IGF-I-MSCs condition when compared to AdGFP-MSCs and vehicle (Fig. 3d,e). Altogether, these data suggest that paracrine mechanisms mediate MSCs modulatory activity on the gene expression profile of hMø.

### MSCs treatment induces the expression of growth factors and matrix metalloprotease-2 in hepatic macrophages

**Fig. 4:**
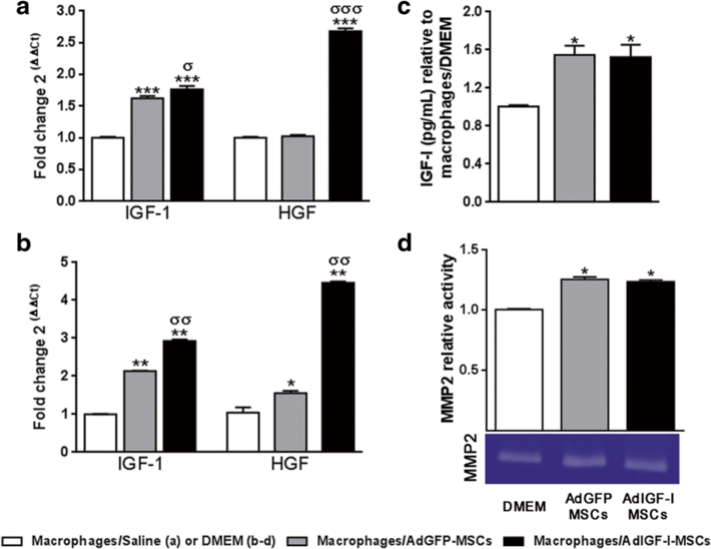
Increased growth factors levels and MMP2 activity in hepatic macrophages after MSCs treatment. Quantitative analyses of IGF-I and HGF mRNA expression levels in macrophages, on in vivo (**a**) or in vitro (**b**) experiments, after saline/DMEM (*white bars*), AdGFP-MSCs (*gray*) or AdIGF-I-MSCs (*black*) pretreatments. **c** IGF-I protein levels in hMø supernatants on in vitro experiments. Macrophages obtained from fibrotic livers were preincubated with conditioned media on in vitro studies. ANOVA Tukey’s post-test, ^*^ *p* < 0.05; ^**^ *p* < 0.01 and ^***^ *p* < 0.001 vs. macrophages/saline (in vivo) or macrophages/DMEM (in vitro); ^σ^ *p* < 0.05, ^σσ^ *p* < 0.01 and ^σσσ^ *p* < 0.001 vs macrophages/AdGFP-MSCs conditions. **d** MMP-2 activity in hMø supernatants after in vitro incubation evaluated by zymography. One representative zymogram is shown. Band intensity of three independent experiments was detected by densitometric evaluation and plotted as MMP-2 relative activity of DMEM/macrophages condition. Dunn’s multiple comparisons test, ^*^ *p* < 0.05 and ^**^ *p* < 0.01 macrophages/DMEM

Considering that macrophages with restorative profile would likely express growth factors, such as IGF-I [19] or HGF, we analyzed their expression levels in hMø at 1 day after MSCs treatments. As shown in Fig. 4a, an upregulation in IGF-I mRNA expression was found in hMø isolated from AdGFP-MSC-treated mice when compared with vehicle, and expression levels of this gene were further increased in the AdIGF-I-MSCs group. Interestingly, the latter was the only condition able to significantly induce HGF expression (Fig. 4a). This result is of significant importance since hepatocytes are known to express receptors for HGF but not for IGF-I [20].

In order to analyze whether factors secreted by MSCs might be involved in IGF-I and HGF upregulation, hMø from fibrotic livers were preincubated with conditioned media from AdIGF-I-MSCs and AdGFP-MSCs. Cells were then washed and collected or maintained in serum-starved medium for additional 24 hours. Consistent with previous results, IGF-I and HGF gene expression levels were significantly higher in hMø exposed to AdIGF-I-MSCs secreted factors when compared to AdGFP-MSCs and DMEM treatments (Fig. 4b), and IGF-I protein levels in hMø supernatants were significantly increased after treatment with MSCs conditioned media when compared to DMEM (Fig. 4c). Overall, these results suggest that systemic administration of AdIGF-I-MSCs induces an upregulation in IGF-I and HGF expression levels in hMø, with involvement of paracrine mechanisms.

Furthermore, hMø preconditioning with MSCs supernatants resulted in an increased MMP2 activity when compared to DMEM condition (Fig. 4d) thus suggesting a restorative phenotype in hMø.

### Conditioned media from hepatic macrophages pretreated with MSCs supernatants reduce HeSCs activation

**Fig. 5:**
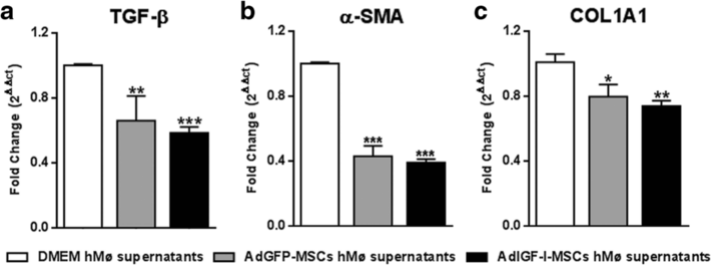
Antifibrotic effects of hepatic macrophages after MSCs treatment. Quantitative analyses of TGF-β1 (**d**), α-SMA (**b**), and COL1A2 (**c**) mRNA expression in hepatic stellate cells after 18 hours incubation with supernatants of hMø after in vitro treatments with DMEM (*white bars*), AdGFP-MSCs (*gray*) or AdIGF-I-MSCs (*black*) conditional medium. ANOVA Tukey’s post-test, ^*^ *p* < 0.05; ^**^ *p* < 0.01, ^***^ *p* < 0.001; ^*^ vs. DMEM hMø supernatants

Based on our results showing the induction of an anti-inflammatory/pro-regenerative profile in hMø shortly after MSCs treatment, we then asked whether factors secreted by macrophages could modulate HeSCs activation. For this purpose, the activated rat hepatic stellate cell line CFSC-2G was incubated 18 hours with conditioned media obtained from hMø preconditioned with MSCs supernatants or with DMEM. CFSC-2G cells were then washed and collected. Interestingly, TGF-β1, α-SMA and COL1A2 mRNA expression levels were downregulated in HeSCs treated with conditioned media from hMø preincubated with MSCs supernatants (Fig. 5a-c). From these data we can now conclude that hMø modulated by MSCs-released factors would likely be involved in the reduction of HeSCs activation previously described in our experimental model.

### Depletion of hepatic macrophages partially abrogates the therapeutic effect of AdIGF-I-MSCs on liver fibrosis in mice

**Fig. 6:**
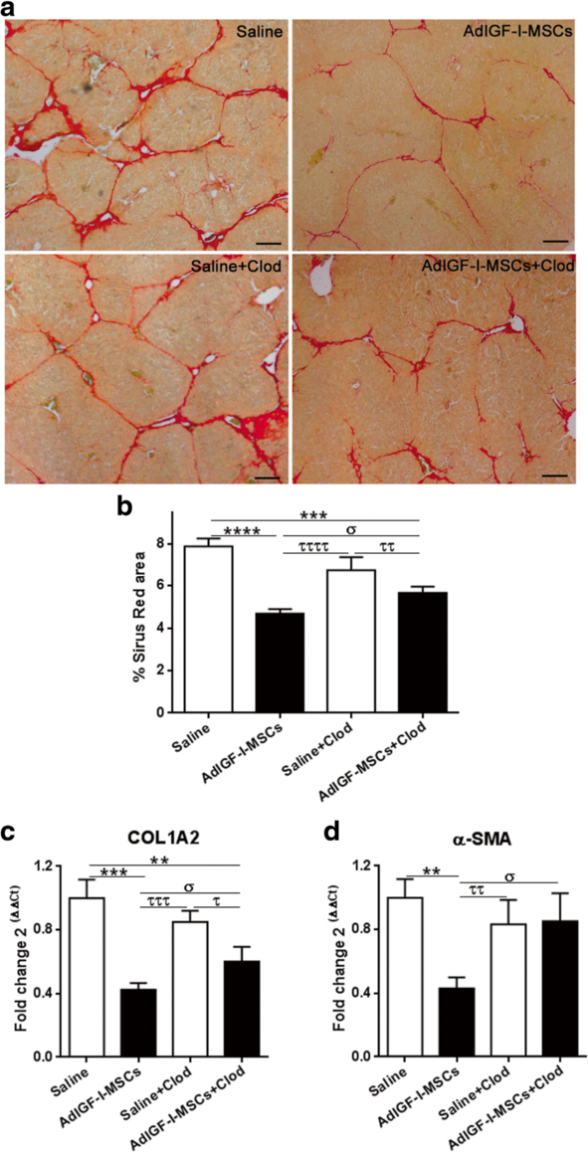
Effect of macrophages depletion in the liver fibrosis amelioration mediated by AdIGF-I-MSCs. **a** Representative images of Sirius Red stained-liver sections from fibrotic mice treated with saline, saline + LipClod, AdIGF-I-MSCs or AdIGF-I-MSCs + LipClod 2 weeks after treatments. **b** Morphometric analyses of Sirius Red-positive area. **c** mRNA expression levels of Col1A2 and **d** α-SMA in liver samples. ANOVA Tukey’s post-test; ^**^ *p* < 0.01, ^***^ *p* < 0.001 and ^****^ *p* < 0.0001 vs. saline; ^σ^ *p* < 0.05 vs AdIGF-I-MSCs + LipClod; ^τ^ *p* < 0.05, ^ττ^ *p* < 0.01, ^τττ^ *p* < 0.001, ^ττττ^ < 0.0001 vs. saline + LipClod

We have herein shown that application of AdIGF-I-MSCs induces a shift in hMø toward an antifibrogenic/pro-regenerative phenotype. We have also shown that macrophages likely play a role in the reduction of HeSCs activation found in our therapeutic model. With these results, we wondered whether hMø might be relevant in the AdIGF-I-MSCs-mediated liver fibrosis amelioration. With this aim, TAA was chronically applied to 6–8-week-old BALB/c mice, three times a week, for 8 weeks. At the sixth week, animals were depleted from hMø by intravenous application of liposome-encapsulated clodronate, or saline as control (not shown). The day after, AdIGF-I-MSCs or vehicle were intravenously injected, and 2 weeks later animals were sacrificed and liver samples analyzed after Sirius Red staining (see Figure S3c). As shown in Fig. 6a-b, depletion of macrophages resulted in a significant abrogation of the therapeutic effect as seen by quantification of collagen deposits. No significant changes were found in liver fibrosis outcome when macrophage-depleted animals were treated with vehicle (control). Consistently, liver COL1A2 mRNA expression levels were found increased in AdIGF-I-MSCs-treated animals injected with clodronate (Fig. 6c). Furthermore, the effect of the therapeutic condition on the reduced activation of HeSCs was abrogated after partial depletion of macrophages as suggested by quantification of α-SMA mRNA expression levels (Fig. 6d). In conclusion, these results implicate hMø in the liver fibrosis amelioration achieved by AdIGF-I-MSCs infusion.

### Role of macrophages in the pro-regenerative mechanisms induced by AdIGF-I-MSCs therapy

**Fig. 7:**
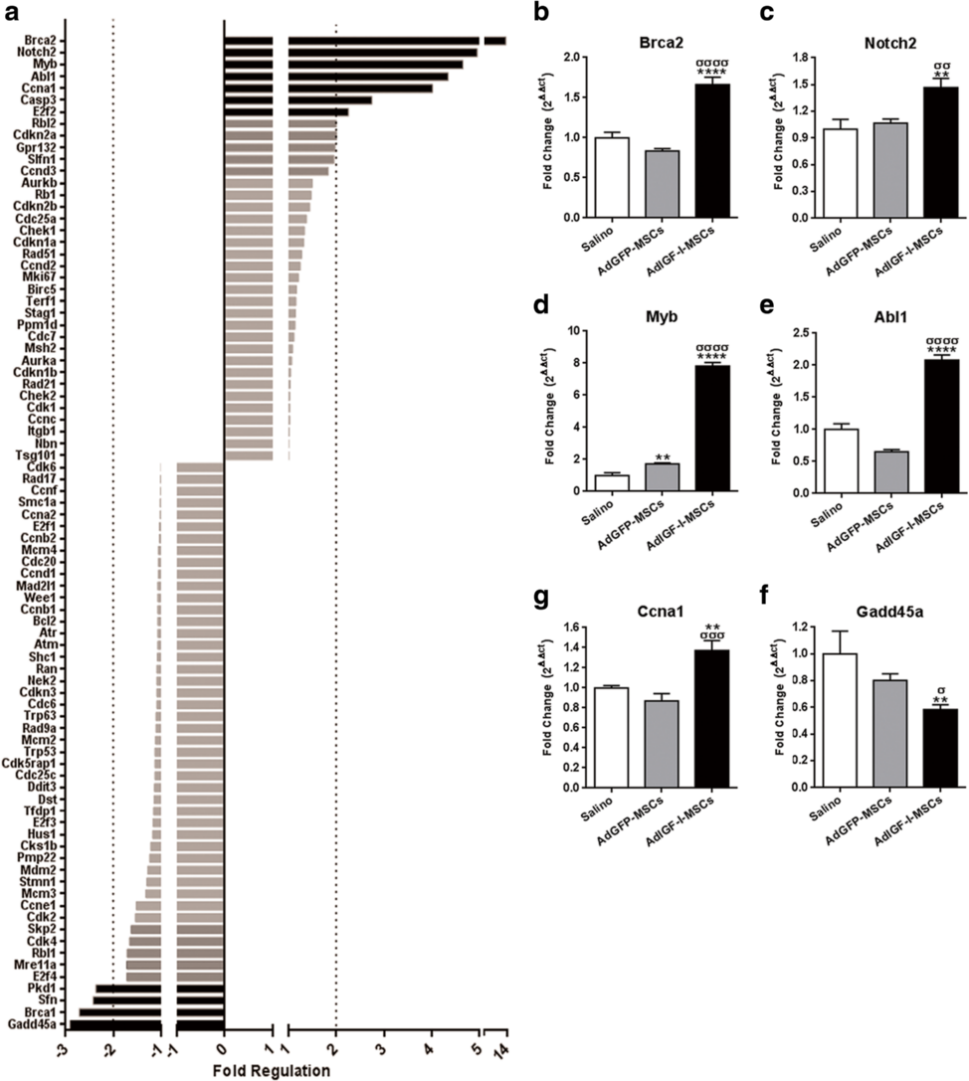
Regulation of cell cycle-related genes by AdIGF-I-MSCs treatment. **a** Screening analyses of mRNA expression profile differences in between fibrotic liver samples after saline (control) or AdIGF-I-MSCs treatments, using a functionally specific qPCR array. *Punctated lines* represent the thresholds; genes with scores above them were considered as differentially regulated (induced: *to the right*; repressed: *to the left*) by AdIGF-I-MSCs. **b**-**g** Changes in the most differentially regulated genes were validated by qPCR, and comparisons against AdGFP-MSCs condition were added. ANOVA Tukey’s post-test, **p < 0.01 and ****p < 0.0001 vs. saline; ^σ^ *p* < 0.05; ^σσ^ *p* < 0.01; ^σσσ^ *p* < 0.001 and ^σσσσ^ *p* < 0.0001 vs. AdGFP-MSCs

We have previously shown that AdIGF-I-MSCs treatment significantly enhanced hepatocyte proliferation, with a peak within 24 hours after cellular application [13]. With the aim of uncovering molecular mechanisms driving the pro-regenerative effect of MSCs, by using PCR array analyses for cell cycle markers, we evaluated the gene expression profile of fibrotic liver pooled samples obtained from AdIGF-I-MSCs and vehicle-treated animals at 1 day after their in vivo application. AdIGF-I-MSCs treatment resulted in the upregulation of seven genes and downregulation of four genes (out of 90 genes; >2 fold change; Fig. 7a). Many of the upregulated genes are involved in DNA repair, such as Breast cancer 2 (Brca2), Myeloblastosis oncogene (Myb), Abelson murine leukemia viral oncogene homolog 1 (Abl1) and G protein-coupled receptor 132 (Gpr132) (fold change: 14; 4.7; 4.3 and 2, respectively; vs. vehicle). Likewise, some of them and others are known to positively regulate cell cycle progression, such as Cyclin A1 (Ccna1), Efr transcription factor 2 (E2f2), Retinoblastoma-like protein 2 (Rbl2) and G protein-coupled receptor 132 (Gpr132) (fold change: 4; 2.4; 2 and 2, respectively). Interestingly, some upregulated genes, such as Myb, Rbl2 and Cyclin-dependent kinase inhibitor 2A (CDKN2A; fold change: 2), code for proteins able to act as tumor suppressors. Among downregulated genes, growth arrest and DNA-damage-inducible 45α (Gadd45α) had the highest score (fold change: 2.9).

In order to validate these results and include data from the AdGFP-MSCs experimental group, individual liver samples were analyzed by qPCR. AdIGF-I-MSCs condition resulted in the upregulation of Brca2, Myb, Abl1, Notch2 and Ccna1 (Fig. 7b-f), and in the downregulation of Gadd45α (Fig. 7g), when compared to AdGFP-MSCs and vehicle conditions. From all these genes, AdGFP-MSCs treatment resulted in a mild increase Myb expression levels with other genes remaining unchanged when compared to vehicle group (Fig. 7b-g).

**Fig. 8:**
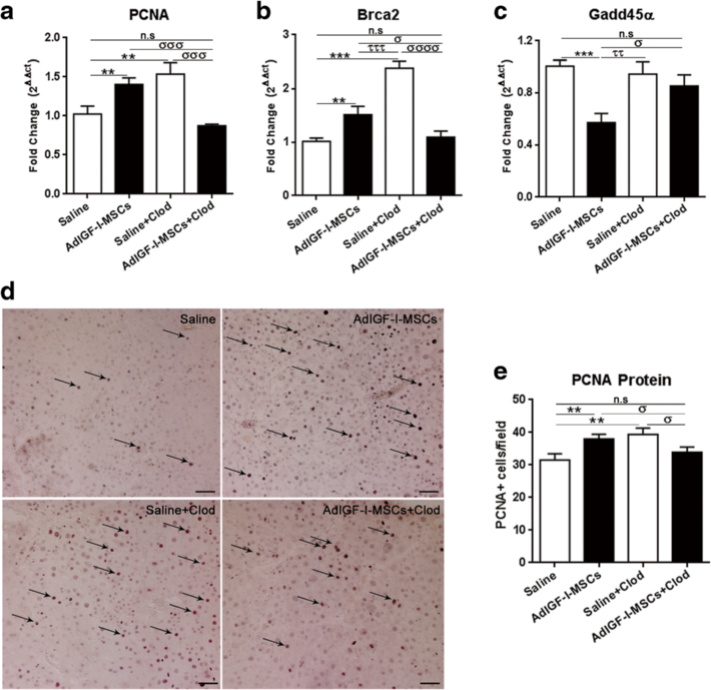
Role of macrophages in AdIGF-I-MSCs induced pro-regenerative mechanisms. PCNA (**a**), Brca2 (**b**), and Gadd45α (**c**) in vivo mRNA expression levels were statistically compared with/without macrophages depletion. **d** Representative microphotographs of PCNA immunostained sections showing changes in proliferation in liver hepatocytes at 14 days after treatments. **e** Statistical comparisons of number of PCNA^+^ cells among groups. ANOVA Tukey’s post-test, ^**^ *p* < 0.01 and ^***^ *p* < 0.001 vs. saline; ^ττ^ *p* < 0.01 and ^τττ^ *p* < 0.001 vs. saline + LipClod; ^σ^ *p* < 0.05; ^σσσ^ *p* < 0.001 and ^σσσσ^ *p* < 0.0001 vs. AdIGF-I-MSCs + LipClod. *n.s* nonsignificant

We then asked whether the depletion of macrophages could abrogate the pro-regenerative and DNA repair effects of AdIGF-I-MSCs treatment. For this, mRNA levels of PCNA, Brca2 and Gadd45α were analyzed by qPCR, 2 weeks after AdIGF-I-MSCs or vehicle administration. The effect of AdIGF-I-MSCs on hepatocytes proliferation and protection was lost when animals were depleted from macrophages, as suggested by changes in the expression of all markers analyzed (Fig. 8a-c). Numbers of PCNA^+^ hepatocytes were also reduced to control vehicle levels thus confirming an essential role of macrophages in AdIGF-I-MSCs-induced proliferation (Fig. 8d, e). In the case of PCNA and Brca2, control vehicle group depleted from macrophages (saline + Clod) showed a very strong pro-regenerative effect; nevertheless, PCNA and Brca2 expression levels were significantly reduced when compared to the saline + Clod control when AdIGF-I-MSCs were applied to macrophages-depleted animals and reached levels which were similar to saline (Fig. 8a,b,d,e). From these results we can conclude that the pro-regenerative mechanisms triggered by AdIGF-I-MSCs are likely dependent on macrophages through hepatocytes protection from DNA damage/DNA repair mechanisms.

## Discussion

We herein show that systemic application of MSCs results in increased numbers of hMø within the first 24 hours. Interestingly, AdIGF-I-MSCs or AdGFP-MSCs treatments differentially regulate hMø gene expression profile. The mRNA expression of pro-inflammatory markers in hMø were found more significantly downregulated after AdIGF-I-MSCs treatment when compared to AdGFP-MSCs one. More importantly, only after AdIGF-I-MSCs in vivo application hMø expressed higher levels of IGF-I and HGF, which suggest an induction of a restorative and pro-regenerative phenotype in hMø. In addition, preincubation with MSCs conditioned media was found to increase MMP2 activity in hMø. These features were shown to be mediated by paracrine mechanisms. Moreover, factors secreted by hMø preincubated with conditioned media from MSCs were seen to reduce HeSCs activation degree. Furthermore, by performing an in vivo systemic macrophage depletion assay, the therapeutic effect of AdIGF-I-MSCs in liver fibrosis was significantly abrogated thus involving macrophages within mechanisms. Finally, results from cell cycle gene screening and data from experiments with macrophages-depleted animals suggest that hMø mediate the early pro-regenerative mechanisms induced by AdIGF-I-MSCs.

Regarding the macrophages phenotype shift induced by MSCs, hMø obtained from AdIGF-I-MSCs were found to upregulate arginase-1 expression levels and downregulate iNOS levels when compared to controls (AdGFP-MSCs and saline). In particular, those changes suggest a reduced local production of NO, which would result in reduced hepatocellular damage and inflammation. Consistently, hMø from MSCs-treated animals showed a downregulation in the expression levels of pro-inflammatory cytokines such as IL-6, IL-1β, IL-12, and TNF-α. Apart from that, a reduction in TGF-β1 mRNA levels was found in hMø from animals treated with AdIGF-I-MSCs, likely involved in the reduced HeSCs activation found later on in livers under such treatment. These in vivo results were mimicked in vitro by incubating hMø obtained from fibrotic livers with MSCs supernatants, involving paracrine mechanisms, although increased hMø IL-10 mRNA expression was observed only in vivo in AdGFP-MSCs-treated mice but not in vitro. Interestingly, at the protein level, IL-10 secretion was increased in the hMø supernatant after preincubation with MSCs conditioned media when compared to DMEM control. Moreover, production of TNF-α and IL-6 protein levels were reduced in the AdIGF-I-MSCs condition when compared to AdGFP-MSCs and DMEM. A little discrepancy between mRNA and protein levels observed could be partially explained by unexplored posttranscriptional and/or translational regulation. A change in macrophages phenotype from a pro-inflammatory M1 to an anti-inflammatory M2 profile was also reported in previous studies. Dayan et al. demonstrated that MSCs induce an increase in the number of CD206^+^ macrophages, and that these cells were involved in cardiac function recovery after myocardial infarction [15]. In some cases, factors mediating this effect were uncovered, such as IL-10, prostaglandin E2 (PGE2) and IL-6, by mean of blockage and inhibitory assays [14, 15, 21]. However, this is the first report involving a switch in hMø induced by MSCs mediating liver fibrosis amelioration.

Overexpression of IGF-I in MSCs by an adenovirus (AdIGF-I-MSCs) was found to further induce antifibrotic mechanisms [13]. Interestingly, macrophages express high levels of IGF-IR [22]. Moreover, both IGF-I, through PI3K/Akt signaling pathway [23], and HGF, through ERK1/2-dependent IL-10 induction [24], were found involved in the M1-to-M2 phenotype change. Thus our results are consistent with the literature suggesting an immunomodulatory role played by MSCs in the phenotype of hMø.

Macrophages involved in liver fibrosis resolution were found to promote extracellular matrix degradation and secrete growth factors able to induce liver regeneration [4, 25, 26]. In addition, Ramachandran et al. identified a subset of macrophages CD11B^hi^ F4/80^int^ Ly-6C^lo^ that could be involved in this resolutive process, which derive from circulating monocytes that infiltrate the liver [25]. In this study, an upregulation in IGF-I expression was observed in hMø from AdGFP-MSCs-treated animals when compared to saline controls, and its levels were further increased in AdIGF-I-MSCs condition. Furthermore, only the latter resulted in a significant upregulation of HGF expression in hMø. Thus, our results together with available literature allow us to conclude that in vivo AdIGF-I-MSCs treatment would induce a functional resolutive phenotype in hMø. Such feature would likely affect HeSC-hMø cross-talk, known to be crucial for liver fibrosis development [3]. Noteworthy, factors secreted by MSCs-preconditioned hMø were shown to likely inhibit HeSCs activation degree. A modulatory effect of MSCs on macrophages phenotype was previously involved in their therapeutic effect in the context of other diseases, such as a myocardial acute infarction experimental model [15], allergic asthma, and renal autoimmunity [14, 27]. More recently by using an acute liver damage experimental model induced by Lee et al. showed evidence of hepatocyte protection by MSCs with similar mechanisms [28]. In the latter study, the authors found that MSCs, trapped into the lungs, were able to induce a M2 profile on alveolar macrophages, with production of high levels of IL-10. In contrast with such results, we previously showed that in the context of liver fibrosis MSCs are mainly recruited to the liver [13, 29]. In addition, we now show that 1 day after MSCs application the phenotype of hMø is changed locally in the liver into a restorative and antifibrogenic one and that hMø likely plays a major role in the therapeutic effect of MSCs, which is enhanced by IGF-I forced overexpression.

IGF and HGF were also previously involved in hepatocyte survival and proliferation [30, 31] and in liver regeneration [32, 33]. Considering that IGF-I and HGF peak at the fibrotic liver tissue a few hours after MSCs treatment, with upregulation of such factors in AdIGF-I-MSCs, hepatocytes as well as in hMø, we have previously speculated on the involvement of hepatocyte proliferation in the experimental beneficial outcome [13]. In our studies, we found a peak of PCNA (a nuclear protein, cofactor of the DNA polymerase during cell division and commonly used as proliferation marker) expression levels and in the number of PCNA+ hepatocytes 24 hours after AdIGF-I-MSCs treatment when compared to controls [13]. PCNA expression levels gradually decreased with time after AdIGF-I-MSCs infusion, although they remained significantly higher than those of control groups at least for 14 days. It is worth noting that a pro-regenerative effect of MSCs has also been shown in different acute and chronic liver damage models [34–36]. Interestingly, while in a chronic liver injury hepatocytes proliferation are the key cells involved in liver regeneration, in an acute damage or hepatectomy liver progenitor cells get also activated, proliferate and are an important source of hepatocytes [37, 38].

With the aim of finding differentially expressed genes involved in hepatocyte-induced proliferation early after MSCs treatment, we performed a qPCR array assay. AdIGF-I-MSCs treatment resulted in the upregulation of genes related to DNA synthesis and repair quality control, such as Brca2, Myb, Abl1, and Gpr132 [39–43]. Other upregulated genes were involved in positive regulation of cell cycle progression, i.e., Ccna1, E2f2, Rbl2, and Gpr132 [44–46]. In addition, Notch2 (a marker of proliferating hepatoblasts [47, 48]) showed higher scores in liver tissue from AdIGF-I-MSCs condition. On the contrary, such treatment was found associated with decreased Gadd45α expression levels, a protein related to cellular stress, increased DNA damage, and cell cycle arrest [49]. Apart from the functions described above, Myb, Cdkn2a, and Rbl2, could also act as tumor suppressor proteins [50]. Importantly, changes in PCNA and in some of the highly regulated genes were lost when animals were depleted of macrophages, thus suggesting that hMø are crucial for the protective and pro-regenerative effects of AdIGF-I-MSCs transplantation. In these last experiments, macrophage-depleted saline controls also showed significant upregulation of PCNA and Brca2 mRNA expression levels, a finding which requires further investigation. Nevertheless such features in this control group seem not to be associated with liver fibrosis amelioration in the TAA model. Our hypothesis is that this effect would likely be caused by removal of pro-fibrogenic macrophages, which releases stress stimuli affecting hepatocytes.

## Conclusions

In conclusion, our results demonstrate for the first time that MSCs transplantation induces a restorative profile of hepatic macrophages, a key event in the reduction of hepatocyte stress, liver regeneration, and fibrosis resolution. Furthermore, this effect could be enhanced by IGF-I overexpression. These data could be useful for future therapeutic developments and give support to the hypothesis that a combination of cell and gene therapy would represent a new potent therapeutic approach to treat liver cirrhosis.

## Additional file

Additional file 1: Figure S1: Experimental design; Materiales and Methods; Table S1: Primers sequences. (ZIP 609 kb)

## Abbreviations

Abl1: Abelson murine leukemia viral oncogene homolog 1
Ad: adenovirus
Brca2: breast cancer 2
Ccna1: cyclin A1
CDKN2A: cyclin-dependent kinase inhibitor 2A
COL1A2: collagen 1A2
DMEM: Dulbecco’s modified Eagle’s medium
E2f2: Efr transcription factor 2
ELISA: enzyme-linked immunosorbent assay
FBS: fetal bovine serum
Gadd45α: growth arrest and DNA-damage-inducible 45α
GFP: green fluorescent protein
Gpr132: G protein-coupled receptor 132
HeSCs: hepatic stellate cells
HGF: hepatocyte growth factor
hMø: hepatic macrophages
i.p.: intraperitoneal
i.v.: intravenous
IGF-I: insulin-like growth factor I
IL-1β: interleukin-1 beta
LipClod: liposome-encapsulated clodronate
MSCs: mesenchymal stromal cells
Myb: myeloblastosis oncogene
PCNA: proliferating cell nuclear antigen
PDGF: platelet-derived growth factor
qPCR: real-time polymerase chain reaction
Rbl2: retinoblastoma-like protein 2
TAA: thioacetamide
TGF-β1: transforming growth factor-beta 1
TNF-α: tumor necrosis factor alpha
TWEAK: tumor necrosis factor-like weak inducer of apoptosis
α-SMA: alpha-smooth muscle actin
