# Supplementary material for: Involvement of hepatic macrophages in the antifibrotic effect of IGF-I-overexpressing mesenchymal stromal cells
Source: Stem Cell Res Ther. 2016 Nov 22;7:172. doi: 10.1186/s13287-016-0424-y (PMC5120504; doi:10.1186/s13287-016-0424-y)
Supplement: Additional file 1: — Figure S1: Experimental design; Materiales and Methods; Table S1: Primers sequences. (ZIP 609 kb) [file 13287_2016_424_MOESM1_ESM.zip › 13287_2016_424_MOESM1_ESM.docx]

**Additional file 1**

**FIGURE LEGENDS**

***Figure S1: Experimental design.***

**(a)** ***In vivo* MSCs effect on hMø.** Fibrosis was induced in BALB/c mice by intraperitoneal (i.p.) administration of 0.2 mg/g bodyweight of thioacetamide (TAA), three times per week, during 6 weeks. Animals were then intravenously injected with saline, AdGFP-MSCs or AdIGF-I-MSCs (5x10^5^cells/animal). The day after saline/MSCs treatments, liver lobes were dissected out and hMø were isolated (first by Histodenz centrifugation and plastic adherence during 20 minutes) for mRNA or protein expression analysis. **(b)** ***In vitro* MSCs effect on hMø.** Fibrosis was induced as described in (a). hMø from fibrotic mice were obtained after six weeks of TAA injection. They were incubated for 18 hours with DMEM (control) or conditioned media (CM) from AdIGF‑I‑MSCs or AdGFP-MSCs. Then, cells were washed and collected for mRNA expression analysis. Alternatively, pre-conditioned hMø were maintaining in culture with DMEM without serum for additional 24 hours and supernatants were harvested for protein levels analysis or hepatic stellate cells (HSCs) treatment. **(c) MSCs effect after hMø** **depletion.** Liver fibrosis was induced in BALB/c mice by chronic administration of TAA, three times per week, during 8 weeks. On week six, hepatic macrophages were depleted by intravenously injection of liposome-encapsulated clodronate or saline solution (non-depleted control mice) (n=12 per group). One day later, saline or AdIGF-I-MSCs were intravenously injected to each group (n=6/condition). At 8 weeks, animals were sacrificed and liver samples were dissected out for subsequent analysis.

**Materiales and Methods**

*Cell culture and adenoviral transfection of mouse bone marrow MSCs*

Male BALB/c mice (6-8 week-old) were sacrificed by cervical dislocation and bone marrow (BM) cells were flushed out from tibia and femur. Mononuclear cells were isolated from bone marrow samples using Ficoll-PaqueTM Plus density gradient (1.077 g/mL; GE Healthcare). Cells were incubated in DMEM low glucose (DMEM lg; Invitrogen/Life Technologies) supplemented with 10% fetal bovine serum (FBS; Gibco/Invitrogen). Medium was replaced 3 days later and cells expanded up to passage 4-8. Recombinant adenoviral vector harboring the rat IGF-I (Ad-IGF-I) and Green Fluorescent Protein (AdGFP) genes (kindly provided by Rodolfo G. Goya, UNLP, Argentina) were used. MSCs were seeded at 70% of confluence in complete medium. Medium was then removed and cells were infected at a multiplicity of infection (MOI) of 30 in DMEM lg and 2% FBS in half of total volume for two hours. After that, medium was completed with 10% FBS in DMEM lg. Otherwise stated, cells were used at three days after adenoviral infection.

*Reverse Transcription-polymerase Chain Reaction (RT-PCR)*

Total RNA (2 µg) were extracted by using Trizol Reagent (Sigma-Aldrich Co.) from homogenized liver tissue or from cells. Total RNA was reverse transcribed with 200U of SuperScript II Reverse Transcriptase (Invitrogen) using 500 ng of Oligo (dT) primers. cDNAs were subjected to real-time polymerase chain reaction (qPCR) (Stratagene Mx3005p, Stratagene). The mRNA levels were quantified by SYBR® Green (Invitrogen). The primers utilized were described in Table 1. All PCR amplifications were carried out using a cycle of 95°C for 10 min and 40 cycles under the following parameters: 95°C for 30 sec, corresponding melting temperature for 1 min, 72°C for 1 min. At the end of the PCR reaction, the temperature was increased from 60°C to 95°C at a rate of 2°C/min, and the fluorescence was measured every 15 sec to construct the melting curve. Values were normalized to levels of glyceraldehyde-3-phosphate dehydrogenase (GAPDH; used as housekeeping) transcript. Data were processed by the ΔΔCt method. The relative amount of the PCR product amplified from untreated animals (saline solution or DMEM condition) was set as 1. A non-template control was run in every assay, and all determinations were performed in triplicates, in two or three independent experiments.

*Enzyme-linked immunosorbent assay (ELISA)*

Cells homogenates or conditioned media were tested in competitive ELISA using kits acquired from R&D Systems to quantify IL-10, IL-12 and IGF-I, following the manufacturer’s recommendations. IL-6 and TNF-α levels were determined using BD OptEIA™ Set Mouse IL-6 and BD OptEIA^TM^ Set Mouse TNF-α (BD Bioscience, CA, USA) following the manufacturer’s recommendations. The protein levels determinations were performed in duplicates, in two or three independents experiments.

*Gelatin Zymography Assay.*

MMPs activity was evaluated by gelatin zymography assay. Briefly 40 μL of supernatant from hMø pre-incubated with AdGFP-MSCs, AdIGF-I-MSCs or DMEM. was run on a 10% SDS_PAGE containing 0.1% gelatin (Sigma-Aldrich). HT-1080 supernatants was run as positive control. The gel was stained with Coomassie Brilliant Blue R-250 for 30 min at room temperature. Gelatinase activity was visualized by negative staining; gel images were obtained with a digital camera (Canon EOS 5D), and were subjected to densitometry analysis using Image J software (NIH, USA). Relative MMP-2 activity was obtained by normalizing values to untreated samples (DMEM).

*Sirius Red Staining.*

Formalin-fixed liver samples were embedded in paraffin and 5 μm sections were stained with Sirius Red for detection of fibrillar collagen. Quantitative analysis of stained area was performed by computerized morphometric analysis. About 50 light microscope images (200X) per specimen were captured and analyzed using the color threshold detection system developed in ImageJ software (NIH, USA). Results were expressed as percentage of positive area.

*Immunohistochemistry for PCNA.*

Paraffin liver sections were incubated with 3% H2O2–methanol for 30 min at room temperature to block endogenous peroxidase. They were then subsequently incubated with avidin and biotin blocking solution (Vector) and incubated overnight at 4°C with a mouse monoclonal anti-PCNA (1/100; Santa Cruz Biotechnology) antibody. After extensive washings, tissue was incubated for 1 hour with peroxidase-coupled biotinylated goat anti-mouse (1/100; Vector) secondary antibody. It was then washed and further incubated with AB complex (Vector). After PBS and 0.1 M acetate buffer washing, samples were incubated with a 3.3-diaminobenzidine (Sigma), ammonium nickel sulfate and H2O2 solution until signal was developed. Primary antibody incubation was omitted in control slides, only rendering a faint staining (not shown). Immunofluorescence protocol was as previously described. Number of liver PCNA positive cells per field was calculated using captured images taken with direct light microscopy (200X; 50 images/condition) and CellProfiler software ([www.cellprofiler.com](http://www.cellprofiler.com/)). It was expressed as mean number of cells/field.
