# Supplementary material for: Involvement of hepatic macrophages in the antifibrotic effect of IGF-I-overexpressing mesenchymal stromal cells
Source: Stem Cell Res Ther. 2016 Nov 22;7:172. doi: 10.1186/s13287-016-0424-y (PMC5120504; doi:10.1186/s13287-016-0424-y)
Supplement: Additional file 1: — Figure S1: Experimental design; Materiales and Methods; Table S1: Primers sequences. (ZIP 609 kb) [file 13287_2016_424_MOESM1_ESM.zip › 13287_2016_424_MOESM3_ESM.docx]

Table S1: Primers sequences

| GENE |  | FOWARDS **(5´-3´)** |  | REVERSE **(5´-3´)** |
| --- | --- | --- | --- | --- |
| ABL1 |  | AGAAAGATGCGCCTGACACC |  | AGGAAGCAGTGGCGATACAG |
| ARGINASE-1 |  | CAGAAGAATGGAAGAGTCAG |  | CAGATATGCAGGGAGTCACC |
| BRCA2 |  | AGCCCAGCTTGAAGCAAGT |  | TGGCTCTTCAGGATCATTCGG |
| CCNA1 |  | AAGCATGCCTCAGTATCCCC |  | CAGGGGAAGAACTACAGGCG |
| COL1A2 |  | CCTACATGGACCAGCAGACTG |  | GGAGGTCTTGGTG GTTTTGTA |
| GADD45A |  | CTGCAGAGCAGAAGACCGAA |  | TACACGCCGACCGTAATGG |
| GAPDH |  | CATCTCTGCCCCCTCTGCTG |  | GCCTGCTTCACCACCTTCTTG |
| HGF |  | GGCTGAAAAGATTGGATCAGG3 |  | CCAGGAACAATGACACCAAGA |
| IGF-I |  | CAGTTCGTGTGTGGACCAAG |  | GTCTT GGGCATGTCAGTGTG |
| IL10 |  | GGTTGCCAAGCCTTATCGGA |  | ACCTGCTCCACTGCCTTGCT |
| IL1B |  | TGACAGTGATGAGAATGACCTGTTC |  | TTGGAAGCAGCCCTTCATCT |
| IL6 |  | AGTTGCCTTCTTGGGACTGA |  | TCCACGATTTCCCAGAGAAC |
| iNOS |  | AAGATGGCCTGGAGGAATGC |  | TGCTGTGCTACAGTTCCGAG |
| MYB |  | CAGATGTGCAGTGCCAACAC |  | GACCAACGCTTCGGACCATA |
| NOTCH2 |  | GTGTGGACAAAGTCAACCGC |  | ATGTCGATCTGGCACACTGG |
| PCNA |  | AAAGATGCCGTCGGGTGAAT |  | TTCCCATTGCCAA GCTCTCC |
| TGF-β1 |  | CCACTCGC TTCTTTGAGACC |  | TAGTGGAAGTGGGTGGGGAC |
| TNFα |  | GACCCTCACACTCAGATCATCTTCT |  | CCACTTGGTGGTTTGCTACGA |
| α‑SMA |  | ACTGGGACGACATGG AAAA |  | CCATCTCCAGAGTCCA GCAC |

­
