# Supplementary figures and images for: Involvement of hepatic macrophages in the antifibrotic effect of IGF-I-overexpressing mesenchymal stromal cells
Source: Stem Cell Res Ther. 2016 Nov 22;7:172. doi: 10.1186/s13287-016-0424-y (PMC5120504; doi:10.1186/s13287-016-0424-y)

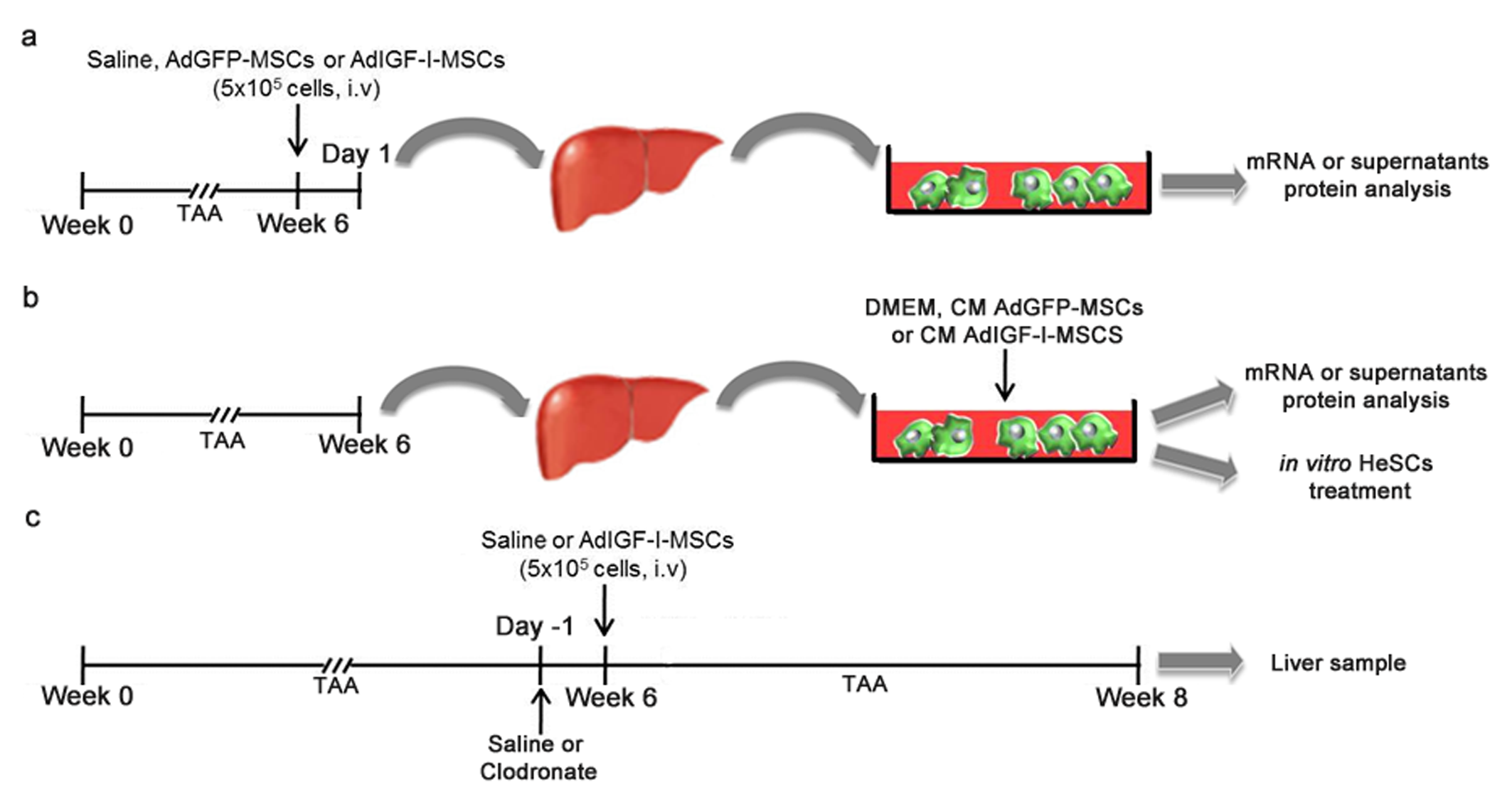

Supplement: Additional file 1: — Figure S1: Experimental design; Materiales and Methods; Table S1: Primers sequences. (ZIP 609 kb) [file 13287_2016_424_MOESM1_ESM.zip › 13287_2016_424_MOESM2_ESM.tif]
